# Mechanisms of antimicrobial resistance in biofilms

**DOI:** 10.1038/s44259-024-00046-3

**Published:** 2024-10-01

**Authors:** Ho Yu Liu, Emma L. Prentice, Mark A. Webber

**Affiliations:** 1 Quadram Institute Biosciences, Norwich Research Park, Norwich, Norfolk NR4 7UQ UK; 2 Norwich Medical School, University of East Anglia, Norwich, Norfolk NR4 7TJ UK; 3 Centre for Microbial Interactions, Norwich Research Park, Norwich, Norfolk NR4 7UG UK

**Keywords:** Antimicrobial resistance, Bacteriology

## Abstract

Most bacteria in nature exist in aggregated communities known as biofilms, and cells within a biofilm demonstrate major physiological changes compared to their planktonic counterparts. Biofilms are associated with many different types of infections which can have severe impacts on patients. Infections involving a biofilm component are often chronic and highly recalcitrant to antibiotic therapy as a result of intrinsic physical factors including extracellular matrix production, low growth rates, altered antibiotic target production and efficient exchange of resistance genes. This review describes the biofilm lifecycle, phenotypic characteristics of a biofilm, and contribution of matrix and persister cells to biofilms intrinsic tolerance to antimicrobials. We also describe how biofilms can evolve antibiotic resistance and transfer resistance genes within biofilms. Multispecies biofilms and the impacts of various interactions, including cooperation and competition, between species on tolerance to antimicrobials in polymicrobial biofilm communities are also discussed.

## Introduction

In nature, most bacteria exist in biofilms, aggregated communities of microorganisms that are encased in a self-produced matrix. Cells in a biofilm exhibit a distinct lifestyle from those in a planktonic state, with strains showing major differences in gene and protein expression when grown as biofilms compared to their planktonic equivalents^1^. The biofilm mode of life is one of the most abundant and robust lifestyles found on earth, and biofilms can be found in seawater, groundwater, soil, and ocean sediment, where they drive the bio-geochemical cycle of many elements in these environments^2,3^. Owing to the protective characteristics of the matrix^4^ and changes in cell physiology that lead to the formation of metabolically dormant cells^2,5^, biofilms are generally highly tolerant of different chemical and physical stressors in the environment^6^. Despite many beneficial uses in industry, biofilms can also pose threats to human health, facilitating the contamination of drinking water^7^ and medical devices, including indwelling implants, contributing to persistent infections that are challenging to eradicate^2,8^. Biofilm infections are particularly problematic as effective treatment is often highly challenging due to the intrinsic resistance to antimicrobials and the innate host immune response^8^. Biofilms are important contributors to many bacterial infections^9^ and are common causes of chronic infections where the prolonged presence of the biofilm induces an adaptive inflammatory response without the biofilm being cleared by the immune system^10–12^. These infections can occur in a range of locations, including in chronic wounds, heart valves and the lungs, as well as on medical implants including catheters and prosthetic devices^9,13^. The impacts of biofilm infections vary but can be very severe. For example, biofilms in the cystic fibrosis (CF) lung underpin chronic infection and are the major reason life expectancy for sufferers is limited to 35–50 years^14^. Chronic wounds caused by biofilms are also a major cause of morbidity, with nearly $300 billion estimated to be spent per year on the management of biofilm wound infections^15^.

### The biofilm life cycle

The unique properties of cells in a biofilm promotes infection and underpins antibiotic resistance. Biofilm formation is an intricate process that involves the production of extracellular components such as adhesins and multiple changes to cell physiology^16^. The specific processes associated with the development of a biofilm and the biofilm structure can vary based on the species and strains of bacteria as well as on the surrounding environmental conditions^16^. For example, *Pseudomonas aeruginosa* biofilms form mushroom-shaped microcolonies in flow chambers when a glucose medium is used, but when citrate is the carbon source, ‘flat’ biofilms are formed^17^. *Staphylococcus aureus* can employ distinct mechanisms for successful biofilm formation depending on the environment. These biofilm archetypes include: the polysaccharide biofilm, which is dependent on the expression of poly-N-acetylglucosamine and polysaccharide intercellular adhesin; the protein/ extracellular (eDNA) biofilm, which uses surface proteins to mediate cell-to-cell contact and incorporate eDNA from lysed cells into the biofilm matrix; the fibrin biofilm, in which fibrin acquired via coagulase-mediated activation of plasminogen, is used as a scaffold to support the biofilm; and the amyloid biofilm, which uses phenol-soluble molecules to both promote biofilm dispersal and accumulation^18^. The programme of biofilm matrix formation used depends on strains and conditions.

Interactions between the host and bacteria strongly impact biofilm formation during infection. For example, *S. aureus* biofilms grown for < 24 h on human plasma-conditioned surfaces, subjected to shear flow in a chemically defined medium to mimic human infection, were significantly more susceptible to rifampicin and vancomycin than biofilms grown on polystyrene in a bacteriological medium^19^.

**Fig. 1:**
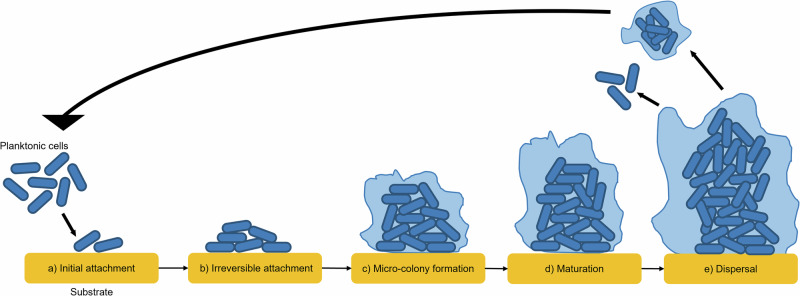
The lifecycle of a surface attached biofilm. Biofilm formation starts with the initial attachment of cells to a substrate (**a**), followed by irreversible attachment of cells (**b**), micro-colony formation (**c**) and biofilm maturation (**d**), and dispersal of cells or aggregates that move on to colonise other substrates (**e**).

Although the mechanisms of biofilm formation are complex, there are some generically important events, and the lifecycle can be described broadly in five main steps: initial attachment, irreversible attachment, micro-colony formation, biofilm maturation and dispersion^1^ (Fig. 1).

The formation of a biofilm commences with the adhesion of free-living planktonic cells to a biotic or abiotic surface^2,20^ (Fig. 1a). Cells can attach to a diverse range of surfaces, including water pipes, indwelling medical devices (e.g. catheters^21,22^), as well as living tissues^23^ (e.g. epithelial cells in the gut and urinary tract^24,25^). Traditional models of biofilm formation have described how single cells initiate binding to a surface where this initial attachment is reversible and followed by committed irreversible attachment^3,22^ (Fig. 1b). We now know initial seeding is often from clumps of cells that represent aggregates of bacteria that can form in vivo, for example, in a mucus layer, or are themselves groups that have been lost from an existing biofilm^26^. During chronic infections, bacteria often attach to each other to form self-contained aggregates that are not associated with substratum^27,28^. It has been suggested that self-contained aggregates may allow bacterial communities to colonise new niches under unfavourable conditions as they are more resilient to stress than free-floating planktonic cells^26^. Furthermore, biofilms can also begin formation via indirect attachment of bacteria to surfaces through attachment to host proteins that coat these surfaces^29^. For example, in infections involving indwelling medical catheters, host fibrin and fibrinogen have been found to promote the attachment of *S. aureus* to the catheters, contributing to biofilm formation^30^. It has been found that the *S. aureus* fibrinogen-binding clumping factor A (ClfA), which binds fibrinogen and fibrin, was key for *coa*-dependent *S. aureus* biofilm formation on plasma-coated surfaces, overall demonstrating an important role for the host in biofilm formation during infection^19^. Recent work has exploited this process to treat staphylococcal biofilm device-related infections under biomimetic conditions where *S. aureus* biofilms exposed to fibrinolytic agents were effectively dispersed, with dispersed cells being killed when antistaphylococcal antimicrobials were added in combination^31^.

Once adhesion of cells has been established (Fig. 1b), the biofilm begins to form microcolonies and enter the maturation step (Fig. 1c, d) in response to signals such as an increase in intracellular cyclic diguanylate monophosphate (c-di-GMP), a secondary messenger molecule^32^ that plays a major role in the regulation of biofilm formation^33^. c-di-GMP is synthesised by diguanylate cyclases (DGCs) and broken down by phosphodiesterases (PDEs), and high levels of c-di-GMP reduce motility and promote a sessile lifestyle. In *Burkholderia cenocepacia*, for example, the protein RpfR has both DGC and PDE activity and mutations in *rpfR* that reduce the activity of the PDE domain to prevent c-di-GMP breakdown, resulting in larger aggregates, increased matrix and biofilm mass production^33^.

During the maturation process, cells expand to form micro-colonies^23^, and the extracellular matrix is secreted^34^ (Fig. 1c, d). The matrix can make up over 90% of the mass of a biofilm^4^ and comprises an agglomeration of various biopolymers, collectively known as extracellular polymeric substances (EPS)^4^. Common biopolymers of the matrix include polysaccharides, lipids, proteins, and eDNA^2,4^. However, the EPS found in a biofilm matrix can vary vastly depending on a range of factors, including which microorganisms are present, nutrient availability, and the environmental temperature^2,4^.

Once the biofilm has matured, cells can detach from the surface and move on to colonise new substrates^35^ (Fig. 1e). The process of cell dispersal is complex^35^, and so far, seeding, erosion and sloughing have been identified as mechanisms of cell dispersal in biofilms^36^. Seeding, also known as central hollowing, is an active process of cell dispersal^36^ in which large quantities of cells or micro-colonies are released promptly from the biofilm, resulting in the formation of hollow cavities within the biofilm^35,36^. This is often initiated by cells in the biofilm in reaction to environmental changes, for example, stress from lack of nutrients or the presence of antimicrobials^22^. In contrast to seeding, sloughing, where substantial fragments detach abruptly from the biofilm^36^, and erosion, where smaller fragments detach from the biofilm over time^35,36^, occur passively as a result of external forces^35^ like mechanical processes such as toothbrushing and shear flow^37^. Recent research has investigated the ability of enzymes, such as glycoside hydrolases, that can break down glycosidic bonds between sugars within the EPS of the biofilm matrix to induce biofilm dispersal in vitro monospecies and multispecies *P. aeruginosa* and *S. aureus* biofilm models have been used to explore whether these enzymes could be used to treat patients with chronic wound infections^38,39^.

### Mechanisms of antimicrobial resistance in biofilms

Some of the fundamental properties of a biofilm described above (metabolic dormancy, protection from EPS) result in intrinsic tolerance to antimicrobials^40^. In addition to this intrinsic tolerance, various features can also facilitate the evolution of antibiotic resistance within and between species of bacteria in a biofilm^41^.

### The biofilm matrix

**Fig. 2:**
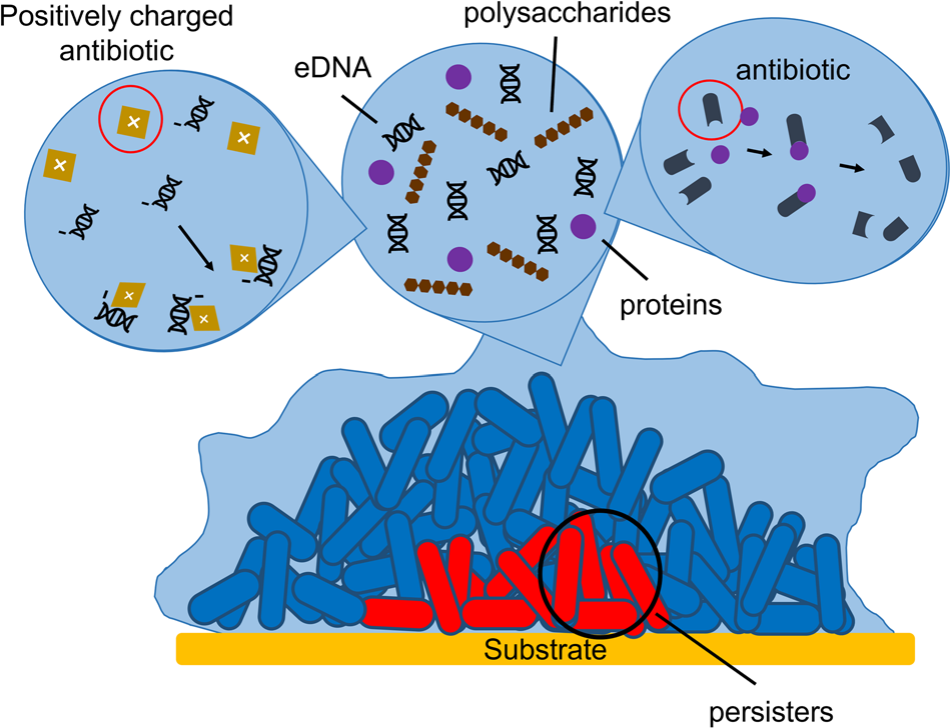
Components of the matrix that can hinder the absorption of antibiotics into the biofilm. Positively charged antibiotics (such as aminoglycosides) can bind to negatively charged eDNA found in the matrix, reducing antibiotic penetration, polysaccharides can present a permeability barrier and secreted enzymes can break down antibiotics resulting in a reduced concentration of antibiotics reaching the bacteria cells^39,42^.

The matrix is a structurally robust layer that acts as a protective barrier for the cells in a biofilm and is a characteristic hallmark of biofilm formation^4,42^. The success of the biofilm lifestyle has largely been attributed to the matrix, and various components of the matrix can have protective properties against a range of environmental stress factors, including antibiotics^42^. The biofilm matrix can hinder antibiotic absorption into the biofilm^40^ (Fig. 2). Some antibiotics form complexes with components of the matrix or are broken down by enzymes, resulting in a reduced concentration of antibiotics reaching the bacterial cells as a consequence^43^, and other antibiotics, such as positively charged aminoglycosides, can bind to negatively charged biopolymers like eDNA^43^ in the matrix, slowing down antibiotic penetration^40^. During chronic infection, polymorphonuclear leukocytes can be recruited to biofilms before undergoing bacteria-induced necrosis, releasing host eDNA, and studies have shown that in the CF lung, eDNA produced by *P. aeruginosa*, together with the host eDNA, can form a physical shield to protect the biofilm from tobramycin and host immune cells^44^. Similarly, *P. aeruginosa* biofilms can also be protected by host neutrophil extracellular trap (NET) formation. In ocular *P. aeruginosa* biofilms, as neutrophils form a layer around the biofilm, toxins released via type III secretion induce NET formation, which surrounds the biofilm and prevents bacterial dissemination but also hinders access of some antibiotics to the biofilm. *P. aeruginosa* susceptibility to tobramycin is greatly decreased when NET formation is induced, and tobramycin becomes unable to clear a biofilm^45^.

In addition to access to a biofilm, the number of target cells present within a biofilm can impact susceptibility to drugs. It has long been known that the density of a target population can impact susceptibility to some antibiotics, a phenomenon known as the inoculum effect^46^. For example, the efficacy of various beta-lactam antibiotics is lower against high-density populations of *Haemophilus influenzae* and *S. aureus*^46,47^. Relatively few studies have explored the importance of the inoculum effect against biofilms; however, research using *P. aeruginosa* biofilms have shown that various beta-lactams, including tobramycin, ceftazidime and imipenem, all demonstrate an inoculum effect against biofilms under laboratory conditions^48,49^.

Apart from impeding the access of antibiotics into biofilms^43^, eDNA found in the matrix also plays an integral role in maintaining the structure of these aggregated microbial communities^5,50^. Although once thought to be unimportant and only released from lysed cells^51^, it has now been acknowledged that eDNA is often essential for the formation and preservation of the biofilm structure^51,52^. eDNA can be produced in considerable amounts through an active process that is linked to outer membrane-derived vesicles in some species of bacteria, including *P. aeruginosa*^51,53^, where the presence of DNase can prevent the formation of biofilms and disperse those that have already formed^51^. This effect of DNase on biofilms has also been observed in other species, including *Escherichia coli* and *Micrococcus luteus*, and NucB, a DNase, was able to disintegrate established biofilms of each species^52^. In addition to DNAse, cellulase has recently been suggested to promote the clearing of biofilms in species such as *E. coli* and *P. aeruginosa* as it breaks down cellulose, an exopolysaccharide present in the biofilm matrix of various species that provides structural protection^54,55^.

The matrix also functions as a reservoir, holding an array of active biomolecules within the biofilm^4,56^. Enzymes found in the matrix can break down complex sugars into fermentable polysaccharides that can be used as a nutrient source^56^ as well as introduce changes to the structure of the matrix to maintain or change the properties of the biofilm^57^. Other proteins in the matrix include amyloids such as curli, which can be important for dictating biofilm structure^58^. Additional biomolecules in the matrix can be derived from the contents of cells that have been lysed^4^, and these cells can release DNA that may become a source of genes for horizontal gene transfer (HGT)^4^. Cells in a biofilm are immobilised and held together closely, allowing for high levels of cell-to-cell interactions making the biofilm an excellent environment for HGT^4,56^ and, therefore, the transfer of antimicrobial resistance (AMR) genes through various routes including conjugation via conjugative plasmids, as well as integrative and conjugative elements^59^, and transduction via bacteriophage^60^, facilitating the role of biofilms as resistance gene reservoirs^61^. Recently, it has also been suggested that outer membrane vesicles (OMVs) may promote the HGT of AMR genes in biofilms of bacterial species, including *P. aeruginosa*^62^.

### Horizontal gene transfer

**Fig. 3:**
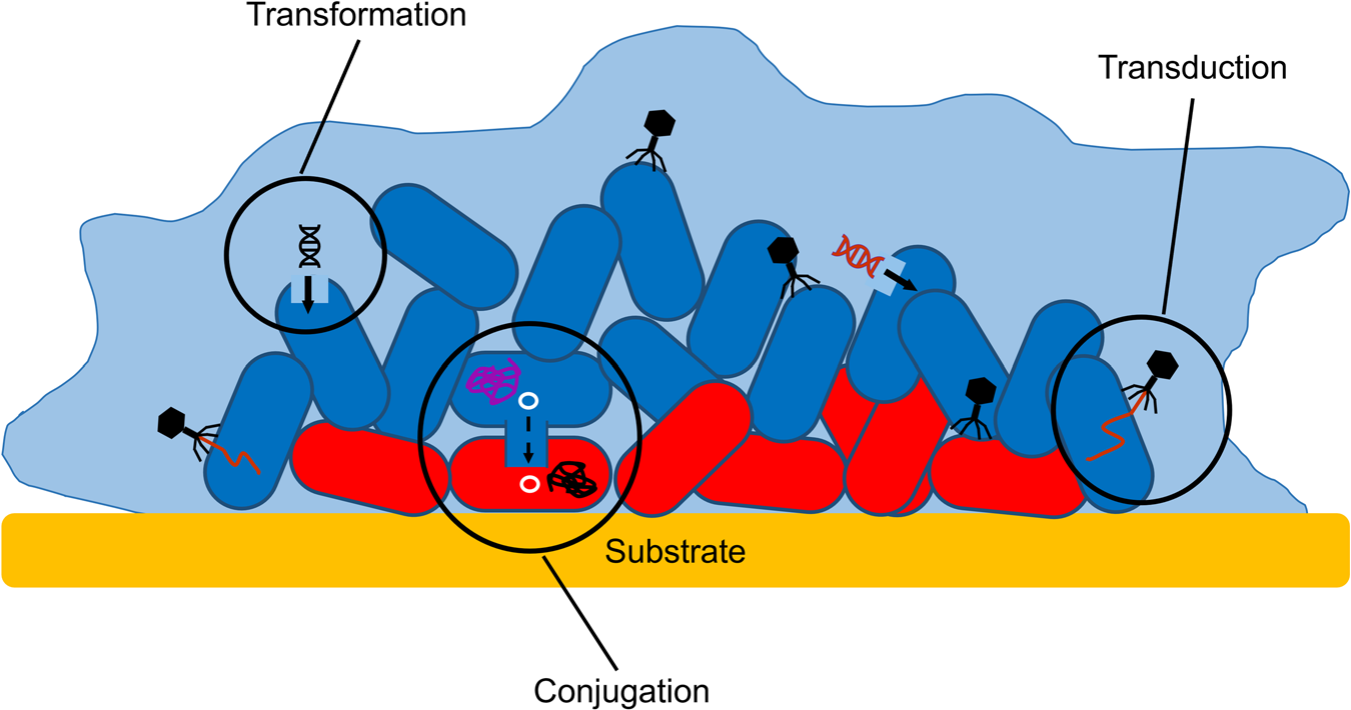
The three main mechanisms of HGT. Transformation, the taking up of DNA from the environment into the bacterial cell, transduction, the insertion of DNA (red) into the bacteria by bacteriophage, and conjugation, the transfer of genes on a plasmid (white) from a donor to a recipient cell through direct contact via pili^64,65^. The bacterial chromosome is shown in pink or black and the plasmid is shown in green.

HGT is a major contributor to the AMR crisis^63^. The emergence and transmission of AMR genes from non-pathogenic to pathogenic bacteria, as well as between different species of pathogenic bacteria, has been fuelled by HGT through the movement of mobile genetic elements (MGEs) carrying genes that confer resistance to most clinically important antibiotics^64^. Genetic material can be transferred between bacteria by HGT, which was traditionally described as consisting of three main mechanisms (Fig. 3): transformation, where DNA from the surrounding environment is taken up by the bacteria; transduction, where the movement of genetic material is facilitated by bacteriophage^65^; and conjugation, where genes are moved between cells through a process that requires direct contact between the donor and recipient cell through structures such as pili that are found on the cell surface^64,65^. All three mechanisms are relevant in biofilms. However, conjugation is often regarded as the most important mechanism for the transfer of AMR genes, particularly in multidrug-resistant Gram-negative pathogens where many genes conferring resistance are carried by conjugative MGEs, including plasmids^64^.

A number of recent studies have proposed other mechanisms of HGT, including lateral transduction and OMV-mediated transfer^62,66^. Lateral transduction is described as the mobilisation of large sections of the bacterial genome by temperate bacteriophage^67^, and thus far, this mechanism has largely been described in *S. aureus* and *Salmonella*^68^. In *S. aureus*, it has been found that many *S. aureus* pathogenicity islands (SaPIs), large mobile gene clusters encoding various accessory proteins and virulence factors^67^, neighbour prophage integration sites, allowing these gene clusters to be transferred via lateral transduction^66^. In *Salmonella*, *Salmonella* pathogenicity islands (SPIs), such as SPI-2, can also be found downstream of prophage attachment sites and be transferred via lateral transduction^66^.

OMVs are nanostructures formed and released from the outer membrane of Gram-negative bacteria^69^ that have various functions, for instance, in cytotoxin and virulence factor transfer as well as nutrient acquisition^70^. More recently, OMVs have also been suggested as a mechanism of HGT^71^, and studies have reported that OMVs may mediate the HGT of plasmids in various species. In *P. aeruginosa*, for example, it was found that OMVs were able to transform *p*BBR1MCS-5, a plasmid encoding for gentamicin resistance, into recipient *P. aeruginosa* cells. Additionally, OMVs obtained from biofilm populations of *P. aeruginosa* were able to transform the plasmid more efficiently compared to those obtained from planktonic populations^62^.

The *bla*_NDM-1_ gene, encoding carbapenem resistance, and *bla*_CTX-M_ genes, encoding extended-spectrum β-lactamases (ESBL)^72^, conferring resistance to cephalosporins, are important examples of AMR genes that have been transferred widely between various pathogenic gram-negative bacteria^63,64^. These can often be readily transferred in biofilms. For example, the movement of *bla*_CTX-M-15_ through a population of *Klebsiella pneumoniae*, causing an outbreak in France, was attributed to the efficient transfer of a plasmid within biofilms^73^.

Although very high concentrations of antibiotics are often needed to kill cells within a biofilm, they have been shown to be highly sensitive to sub-inhibitory concentrations of drugs, which can rapidly select for mutants with resistance mutations^5^. Evolution of AMR occurs due to both the acquisition of point mutations as well as HGT and adaptation may result in changes to other phenotypic traits of the bacteria, including the ability to form biofilms^5^. For example, *Salmonella* biofilms were shown to rapidly evolve resistance when exposed to sub-lethal concentrations of either ciprofloxacin, cefotaxime or azithromycin. Whilst resistance emerged rapidly, mutants were significantly less able to form a biofilm, demonstrating tradeoffs in adaptation^5^. In addition to antimicrobials, studies have shown that biofilm evolution can also be driven by non-antibiotic antimicrobials, including toxic metals like copper^74^, and these toxic metals have been proposed to promote the spread of resistance in biofilms through HGT^74^.

Compared to cells in the planktonic state, HGT occurs much more frequently between cells in a biofilm community^75^, and it has been identified that the rate of conjugation can be increased by up to 16000-fold in *S. aureus* biofilms compared to their planktonic equivalents^76^. There are several reasons proposed for this, including the close proximity of cells within a biofilm that allows for efficient intercellular communication^4^ and the large reservoir of diverse DNA and AMR genes present within a polymicrobial biofilm^61^. The importance of HGT for the transmission of AMR genes in biofilms has been demonstrated in oral biofilms, where mutated mosaic *pbp2x* genes can be transferred between different *Streptococcus* spp, resulting in penicillin resistance^77^.

The formation of biofilms has also been found to facilitate plasmid persistence^76,78^ in the absence of selection, with examples where plasmid maintenance is much higher in biofilm populations relative to planktonic counterparts^79^. Persister cells, which are common in biofilms, can act as plasmid reservoirs where host cells survive antibiotic challenge^80^. This has been demonstrated for *Salmonella enterica serovar* Typhimurium in mice, where persisters harbouring AMR plasmids can survive antibiotic treatment before then being able to efficiently spread AMR through conjugation to other bacteria, such as *E. coli*, in the gut microbiota^80^.

The ability to maintain AMR plasmids, in combination with the elevated levels of HGT in biofilms, has been suggested as an important mechanism contributing to the evolution and spread of resistance in pathogenic microbes^76^, a major cause for concern given the role biofilms play in persistent, chronic infections^61^. Away from the clinical environment, many food-associated biofilms are multispecies and demonstrate higher resistance to disinfectants compared to monospecies biofilms^81,82^. The intrinsic ability of biofilms to tolerate biocides leads to persistent contamination of environments in the food chain, encouraging plasmid stability and HGT^83,84^. This can be exacerbated by other stresses in the food processing environment, such as high salt concentrations and low temperatures, which can alter conjugation rates and thereby influence the spread of resistance through the HGT of plasmids carrying AMR genes, resulting in reservoirs of AMR biofilms in the food chain which can result in contamination of products^85^.

### Tolerance and persistence

The ability to survive antibiotic exposure can be conferred by the carriage of a specific gene or mutation, which renders a target cell resistant to an antibiotic. However, physiological changes to a cell’s metabolism can also be important in determining survival in the presence of an antibiotic. Within a biofilm, there are cells present at various phases of the growth cycle, with metabolically active cells generally being found at the surface of the biofilm^86^ and dormant, slow-growing cells, as well as metabolically inactive cells, including ‘persister’ cells^87^, largely being found in the deeper layers^8,40^. Slow-growing cells often display ‘tolerance’ to stress, including antibiotics. Tolerance is characterised by an ability to survive temporary exposure to concentrations of antibiotics that would typically be fatal^88^. This is a distinct phenotype from persistence which is usually exhibited by a smaller subpopulation of persister cells which have entered a distinct dormant state where growth is fully arrested^89^.

**Fig. 4:**
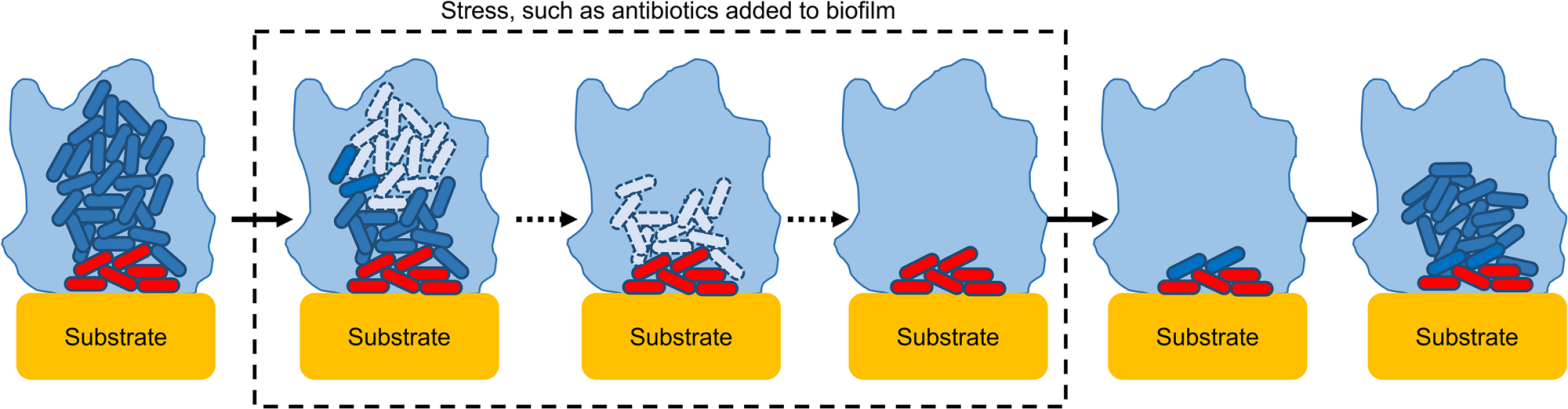
Heterogeneity of susceptibility of cells within a biofilm to antibiotics. The repopulation of a biofilm infection by persisters (red) after actively growing biofilm cells are killed by stress such as antibiotics.

Persister cells undergo a phenotypic, rather than genetic, change into a state of metabolic inactivity^87,90^. These cells are commonly described as having restricted synthesis of macromolecules^5^, arrested growth^40^, and an ability to tolerate a wide range of antimicrobials, particularly those that are bactericidal^90^. Many antimicrobials target cells that are actively growing and replicating^8^, and the presence of persisters may interfere with the action of antimicrobials as the cellular processes they target are no longer crucial for the survival of these cells^5^. Persister cells contribute considerably to the chronic nature of biofilm infections as the site of infection can be repopulated by persisters after the cells sensitive to antimicrobials are eliminated and treatment is ceased^8,40^ (Fig. 4). Regular treatment using antibiotics has been shown to lead to an increase in infections comprising resistant strains of bacteria due to the selection of resistance in vivo, and studies have proposed that the reservoir of persistent cells contributes to this^91^.

The exact mechanisms of persister formation are not fully understood, and relatively few species have been studied^86^. However, the generation of large quantities of persisters in biofilms have been connected with a number of toxin-antitoxin (TA) systems^40^, and certain stress conditions have been shown to increase the rate of persister cell formation^43,44^. The *E. coli hipAB* TA system is a well-studied system associated with the formation of persisters^92^. In the *hipAB* TA system, the HipA toxic protein phosphorylates Glu-tRNA synthetase, which suppresses protein synthesis in the cell^92,93^; this can be neutralised by the antitoxin HipB via the formation of a complex which inhibits HipA transcription^94^. Stress from various stimuli in the environment, such as DNA damage, antimicrobials, and starvation^40,94^, has been shown to lead to stress-related expression of toxins in TA systems^93^, resulting in protein synthesis suppression, and this has been suggested to result in elevated levels of persisters^94^.

Although tolerance, persistence and resistance are distinctly different bacterial states, they are not mutually exclusive and are often interconnected. For instance, persisters can promote the selection of resistant strains of pathogenic bacteria due to their association with chronic, recurrent infections that require the prolonged use of antimicrobial treatment^95,96^, and tolerance has been suggested to increase the rate of which resistant bacterial strains are evolved^97^. Therefore, given the role that they play in the development of AMR in bacteria, it is important to understand and study resistance in conjunction with tolerance and persistence in order to address AMR as a whole^98^.

### Interactions between cells within a biofilm and antimicrobial resistance

Most biofilms found in nature are polymicrobial^10^, including biofilms associated with infections in humans, for instance, in the CF lung and the oral cavity or in chronic wounds^99^. Despite this, most research in the past has been conducted using monospecies planktonic cultures that do not accurately reflect real-world bacterial communities^5,100^. More recent studies have developed tools to investigate the complexity of multispecies biofilms.

Cells in a biofilm are held within close proximity of each other by EPS in the matrix, enabling strong cell-to-cell interactions to occur between them^4^. These interactions are critical and govern the spatial organisation of strains to induce cooperation or competition in biofilms^101^. Various interspecies interactions in polymicrobial communities have been shown to modify antibiotic efficacy, resulting in other members of the community being less susceptible to treatments during polymicrobial infections^100^. The closed environment provided by the biofilm matrix also helps to promote intercellular signalling through mechanisms such as quorum sensing as well as establish synergistic cooperation between the cells^2^.

Synergy has been shown to occur during the formation of multispecies biofilms when stress factors, such as the addition of biocides or removal of nutrients, have been implemented in the environment^102^, and previous studies have suggested that biofilm formation and resistance to antimicrobials can be promoted by synergistic interactions in multispecies biofilms^103^. An example is where *streptococcus* spp. in the oral cavity interacts synergistically with *Candida albicans* in a multispecies biofilm. *C. albicans* can increase biofilm formation in streptococci, and in turn, the streptococci can increase the invasive characteristics of the fungi^104^.

Stress within a biofilm can come from limited space, nutrient availability, the presence of metabolic waste products or from external sources such as biocides and antimicrobials. The adaptive nature of cells in response to stress can promote interactions between species in the form of competition and cooperation and can lead to the formation of persister cells and lower susceptibility to antimicrobials within the biofilm^2,40,96^.

### Quorum sensing

Quorum sensing is the regulation of gene expression in response to changes in the density of a bacterial community^105^. Quorum sensing allows cells in a biofilm to coordinate behaviours^106^ and is mediated through the production and detection of bacterial chemical signal molecules known as autoinducers^99^. Quorum sensing can be responsible for the regulation of various bacterial processes, including the expression of virulence factors^107^. It has been demonstrated that quorum sensing plays a role in infections caused by *P. aeruginosa*, for example, in the CF lung, where mRNA transcripts for *lasR* and *lasI* (genes involved in the *P. aeruginosa* quorum sensing) have been found in mucus samples obtained from CF patients. A decline in virulence is observed in *P. aeruginosa* when there is a deficiency in components involved in quorum sensing^108^. Quorum sensing also controls biofilm formation^107^, and studies have shown that suppressing quorum sensing in bacteria can impede biofilm formation^109^.

As well as being important in coordinating community behaviour within biofilms, quorum sensing has also been shown to impact the antibiotic susceptibility of biofilms. Quorum sensing can elevate bacterial resistance to various stressors, including oxidative, heavy metal and thermal stress, stress from the immune system, and stress from antibiotics such as tobramycin^110^. Studies have investigated the potential of combining antibiotic therapy with quorum sensing inhibitors when treating *P. aeruginosa* and *S. aureus* biofilms^111^. In *P. aeruginosa* and *S. aureus* biofilm wound models, the use of quorum sensing inhibitors resulted in increased susceptibility of the biofilm to the antibiotics tested. This was also observed in *Caenorhabditis elegans* and *Galleria mellonella* models, where a significantly larger number of infected *C. elegans* and *G. mellonella* survived when treated with both quorum sensing inhibitors and antibiotics compared to those that were just treated with antibiotics. These studies show that quorum sensing plays a role in the resistance of bacteria to various antimicrobials, and treating biofilm infections with a combination of quorum sensing inhibitors and antibiotics may lead to higher treatment success rates in the future^111^.

A number of quorum sensing pathways, distinguished by the type of autoinducer involved, have been identified^99^, and it has been found that some pathways, such as the autoinducer-2 (AI-2) pathway, found broadly across both Gram-positive and Gram-negative species of bacteria, can mediate interspecies communication^99,112^. This system plays a key part in the establishment of multispecies biofilms^113^, for example, in biofilms comprising of *H. influenzae* and *Moraxella catarrhalis* in rodent otitis media infections. Although AI-2 could not be produced by *M. catarrhalis*, AI-2 is produced by *H. influenzae* which influences *M. catarrhalis* to produce more biomass with biofilms becoming consequently less antibiotic susceptible^112^.

### Competition between bacterial species within a biofilm

Bacteria occupying a similar niche can interact with each other in various ways, which can result in synergy or antagonism. Whilst many species are indifferent to the presence of others, many can impact others in a way which results in competition between them^99^. Mechanisms of competition in biofilms can be split broadly into two groups: exploitative competition, an indirect mechanism where a species of bacteria hinders another species’ access to nutrients or resources; and interference competition, where the survival of a species is directly affected by mechanisms such as the secretion of growth inhibitors like antibiotics by its competition^114^, as well as by the production of molecules that can prevent the attachment and colonisation of new species in the biofilm^115,116^. These competitive interactions are essential for the evolution and shaping of multispecies biofilms^99^, and studies have suggested that competitive interactions can increase tolerance to antimicrobials in multispecies biofilms^117^.

Cells within a biofilm can protect themselves from the stress of competitors passively rather than antagonistically. In *S*. Typhimurium, the presence of competing strains and species can result in increased biofilm production and antibiotic tolerance. It has been demonstrated that in the presence of *E. coli*, a genetically distinct *S*. Typhimurium strain upregulated genes involved in biofilm formation, efflux, invasion of host cells, and antibiotic tolerance^118^. Genes upregulated in the presence of competition included the *aadA* gene, which encodes an aminoglycoside adenylyltransferase involved in resistance to aminoglycosides, such as streptomycin and spectinomycin^119^. Additionally, the *tolC* gene, encoding the outer membrane component of the AcrABTolC efflux pump, was also upregulated in mixed species biofilms, suggesting increased efflux of antimicrobials including quinolones, chloramphenicol, and tetracyclines occur in mixed species biofilms^118^. The efflux of antimicrobials by efflux pumps can lead to sub-inhibitory intracellular concentrations of drugs, which can promote the selection of AMR strains of bacteria^120^.

### Cooperation between bacterial species within a biofilm

Whilst competition can be antagonistic, there are also many examples of cooperation within a biofilm, where cells can behave collectively, providing them access to the benefits from behaviours which would not be possible for individual cells on their own^121^.

Some species of bacteria are capable of cooperating via coaggregation^99^, a process that requires highly specific interactions between pairs of bacteria^122^ and is essential for the formation of multispecies biofilms^123^. Coaggregation allows different species to attach to one another to stabilise the biofilm and protect all species involved^124^. An early example of bacterial coaggregation arose from investigations into dental plaque obtained from the human oral cavity^123^. Biofilms in the oral cavity can develop sequentially, where species of bacteria such as *Streptococcus mutans* and *Streptococcus gordonii* can colonise the surface of teeth first, altering the environmental conditions that then allow a succession of other species of bacteria to colonise the surface^99^. Coaggregation using curli produced by many Gram-negative species is important in the gastrointestinal tract, and cross-seeding of curli subunits between species of the gut microbiota increases surface attachment of cells and facilitates biofilm formation^125^. For example, it was demonstrated that curli expression was associated with enhanced biofilm formation and tolerance to common biocides in a range of Shiga toxin-producing *E. coli* strains^126^. Furthermore, in *E. coli* or *S*. Typhimurium strains lacking EPS expression, a significant increase in tolerance to biocides was observed when these strains formed a mixed-species biofilm with an EPS-producing companion, compared to when grown in a monoculture. This demonstrates how in mixed communities, common goods can be exploited by individual strains, and this can affect antimicrobial susceptibility^126,127^.

A study of interactions between isolates of *E. coli*, *P. aeruginosa*, and *Enterobacter cloacae* from water sources and the ability of chlorine to eradicate monospecies found that within multispecies biofilms, a chlorine concentration of 50-300-fold higher than for monospecies biofilms was required^128^. Enhanced tolerance to disinfectants was also found in *Listeria monocytogenes* and *Lactobacillus plantarum* multispecies biofilms^129^. A study also found that when grown as monospecies biofilms, *P. aeruginosa, Pseudomonas protegens*, and *K. pneumoniae* were more susceptible to SDS and tobramycin^130^, whereas multispecies biofilms were resistant to both agents. *P. aeruginosa* encodes a secreted SDS hydrolase (SdsA1), which can degrade and metabolise SDS within the biofilm. Additionally, *P. protegens* produces aminoglycoside-modifying enzymes that break down tobramycin and offer a community benefit. When the three species were grown as monocultures and exposed to tobramycin, only *P. protegens* survived, showing the importance of different roles within a multispecies biofilm and how important it is to know which species are present as a species may enjoy resistance to an antimicrobial without possessing a specific resistance mechanism itself. Furthermore, it was found that species common in CF patients have higher biomass and less susceptibility to a variety of antibiotics—including tobramycin, ciprofloxacin, cefotaxime, and chloramphenicol—when grown in a multispecies biofilm with *P. aeruginosa*^131^.

## Conclusions

The majority of bacteria exist within biofilms^5^, a context where various phenotypic characteristics contribute to the elevated levels of tolerance to antimicrobials observed compared to their planktonic equivalents^5,40^. The high levels of cell-to-cell interactions in biofilms make these communities an excellent environment for the evolution of AMR through HGT^4,61^. Given that most biofilms, including those that are associated with the majority of infections in humans, are polymicrobial^5,9,10^, it is important to recognise that bacteria may behave differently when in a multispecies community^100^ and investigate the evolution of AMR in bacteria in a biofilm context^5^. Furthermore, although it is well established that HGT plays a crucial role in the spread of bacterial resistance, the direct correlation between the use of antimicrobials and the impact on the rate of HGT is poorly understood^132^, and there remains a lack of understanding of the mechanisms and factors driving plasmid movement in multispecies biofilms^5,100^. Interspecies interactions in polymicrobial communities can modify antibiotic efficacy, resulting in members of the community being less susceptible to treatments during polymicrobial infections^100^. In the future, developing models to study and understand AMR using models of mixed community biofilms will be needed to better understand how bacteria survive and how AMR evolves in this crucial context. How environmental stresses can exacerbate and influence rates of HGT in biofilms should be explored and alongside conjugation, the possible roles of OMVs and lateral transduction as mechanisms of HGT in biofilms should also be studied. It would also be useful to build on current research and further investigate the genes that are crucial for biofilm formation, in addition to those that drive HGT of resistance genes in biofilms as this knowledge will be required in the development of future strategies to treat and manage biofilm infections, as well as control the spread of AMR in bacterial populations.
